# Rapid Testing May Not Improve Uptake of HIV Testing and Same Day Results in a Rural South African Community: A Cohort Study of 12,000 Women

**DOI:** 10.1371/journal.pone.0003501

**Published:** 2008-10-23

**Authors:** Ntombizodumo B. Mkwanazi, Deven Patel, Marie-Louise Newell, Nigel C. Rollins, A. Coutsoudis, H. M. Coovadia, R. M. Bland

**Affiliations:** 1 Africa Centre for Health and Population Studies, University of KwaZulu-Natal, Durban, South Africa; 2 Centre for Paediatric Epidemiology and Biostatistics, Institute of Child Health, University College London, London, United Kingdom; 3 Department of Paediatrics and Child Health, University of KwaZulu-Natal, Durban, South Africa; 4 Centre for HIV/AIDS Networking, University of KwaZulu-Natal, Durban, South Africa; 5 Division of Developmental Medicine, University of Glasgow, Glasgow, United Kingdom; University of Connecticut, United States of America

## Abstract

**Background:**

Rapid testing of pregnant women aims to increase uptake of HIV testing and results and thus optimize care. We report on the acceptability of HIV counselling and testing, and uptake of results, before and after the introduction of rapid testing in this area.

**Methods and Principal Findings:**

HIV counsellors offered counselling and testing to women attending 8 antenatal clinics, prior to enrolment into a study examining infant feeding and postnatal HIV transmission. From August 2001 to April 2003, blood was sent for HIV ELISA testing in line with the Prevention of Mother-to-Child Transmission (PMTCT) programme in the district. From May 2003 to September 2004 women were offered a rapid HIV test as part of the PMTCT programme, but also continued to have ELISA testing for study purposes. Of 12,323 women counselled, 5,879 attended clinic prior to May 2003, and 6,444 after May 2003 when rapid testing was introduced; of whom 4,324 (74.6%) and 4,810 (74.6%) agreed to have an HIV test respectively. Of the 4,810 women who had a rapid HIV test, only 166 (3.4%) requested to receive their results on the same day as testing, the remainder opted to return for results at a later appointment. Women with secondary school education were less likely to agree to testing than those with no education (AOR 0.648, p<0.001), as were women aged 21–35 (AOR 0.762, p<0.001) and >35 years (AOR 0.756, p<0.01) compared to those <20 years.

**Conclusions:**

Contrary to other reports, few women who had rapid tests accepted their HIV results the same day. Finding strategies to increase the proportion of pregnant women knowing their HIV results is critical so that appropriate care can be given.

## Introduction

Pregnant women need to know their HIV status to receive optimal care during pregnancy, delivery and postnatally[1], [2], [3]. Antenatal rapid testing aims to increase efficiency at clinics by avoiding transportation of samples to laboratories; increase the proportion of women receiving same-day results; and ensure that women booking late in pregnancy obtain HIV results prior to delivery[4]. However, despite the widespread introduction of programmes to prevent mother-to-child transmission (MTCT) of HIV, many women decline HIV testing for reasons that are not fully understood[1], [5], [6], [7], [8], [9], [10], [11], and women who are tested do not always wish to know their results. Reports of increased uptake of HIV results with Rapid Tests (and immediate results)[12] appear counter-intuitive, and may reflect compliant behaviour rather than valid consent.

We report on the acceptability of HIV testing and returning for results, in a cohort of pregnant women from a rural area of South Africa with one of the highest HIV prevalences in the world[13], [14]. The women were part of a large study examining the risks of postnatal HIV transmission associated with different modes of infant feeding[15], [16], which started enrolment at the same time as a Prevention of Mother-to-Child Transmission (PMTCT) programme was implemented in the area. The findings reported represent an operational setting, and the paper evaluates an evolving programme and discusses what the results might mean.

## Methods

Pregnant women attending 8 clinics in rural KwaZulu-Natal were offered HIV voluntary counselling and testing prior to enrolment into a cohort study investigating infant feeding and HIV transmission[15]. Local government clinics are organized to render antenatal care, with HIV counselling and testing, on specific days of the week. To cope with large client numbers, a 3-stage group counselling process was employed at all the clinics in the area (14 fixed government clinics at the time of the study).

### Stage 1 (20 minutes) Group Education

Clinic assistants conducted a group education session to all women (10–60 per session) waiting at the antenatal clinic. Topics covered included: general HIV/AIDS information, definition of disease, transmission modes, mother-to-child transmission issues, advantages and disadvantages of testing, interpretation of positive, negative and indeterminate results.

### Stage 2 (15 minutes) Group Counselling

HIV counsellors conducted small group counselling with five to six women in a private room. They addressed issues of confidentiality, personal risk assessment, exploration of women's support systems, and interpretation of results. Clients who may have been hesitant to ask specific questions in a larger group had chance to voice their concerns at this stage.

### Stage 3 (5 minutes or longer, depending on the individual woman) Individual Counselling

Women were seen individually by the HIV counsellor and offered pre-test counselling. Any personal issues were discussed. Consent for testing was obtained at this stage.

Lay HIV counsellors, who had completed 12 years of schooling, were selected following assessments of literacy, numeracy and basic counselling skills. They completed a standard 10-day HIV/AIDS counselling course and the World Health Organization training courses in HIV and infant feeding[17] and breastfeeding[18]. They received regular training and mentorship throughout the period of the study. The supervision was provided by senior counsellors and psychologists, who ‘sat in’ on counselling sessions with individual counsellors once a month, and conducted group in-service sessions with all the counsellors at least once per month.

From August 2001 to April 2003, dried blood spots were sent for laboratory HIV ELISA testing with results available two weeks later. From May 2003 to September 2004, rapid testing was introduced and women were offered an immediate definitive rapid result, in addition to blood sent to the laboratory for ELISA testing. Univariable comparisons for categorical variables were assessed with a χ^2^ test or χ^2^ for linear trend. Odds ratios (OR) and adjusted odds ratios (AOR) were obtained from univariable and multivariable logistic regression models, respectively. Analyses were performed using SPSS version 12 and R version 2.2.0.

Prior to August 2001 there was no PMTCT (Prevention of Mother to Child Transmission) programme in the district. Furthermore, highly active anti-retroviral treatment (HAART) was not routinely available through the health services at the time of the study. However, all pregnant women were routinely offered 5 mg of folate daily, 200 mg of ferrous fumarate from the first antenatal visit until delivery, and 200,000 IU of vitamin A after delivery, and all infected women and their infants were offered single-dose nevirapine[19]. Clinical follow-up for all women and children in the study was given by study staff from enrollment to two years post-delivery. A treatment programme has since been established in the district in September 2004, and women identified as HIV-infected in the study have been referred for assessment for antiretroviral treatment.

The findings from the cohort of pregnant women reported represent part of a wider programme of research in the same geographical location. A demographic surveillance system operates in the same area, established in the year 2000[20]. In 2003 population-based HIV testing through annual surveys was started in the surveillance area and shows some of the highest population-based infection rates ever documented world-wide; prevalence of 51% (95% CI 47–55%) amongst women aged 25–29 and 44% (95% CI 38–49%) in men aged 30–34[20]. Of the 25,901 people resident in the area and eligible for inclusion in the population-based HIV survey (women aged 15–49 years and men aged 15–54 years), 19,867 (77%) were contacted for HIV testing, of whom 11,551 (58%) consented to be tested[20]. However, less than 5% of those tested returned for their results (personal communication, Till Barnighausen, Africa Centre). Whilst the latter results are from a population surveillance system and not a clinic setting providing voluntary counselling and testing, they do provide greater contextual information with which to interpret the results presented in this manuscript.

This study was approved by the Biomedical Research Ethics Committee of the University of KwaZulu-Natal, Durban, South Africa.

## Results

**Figure 1:**
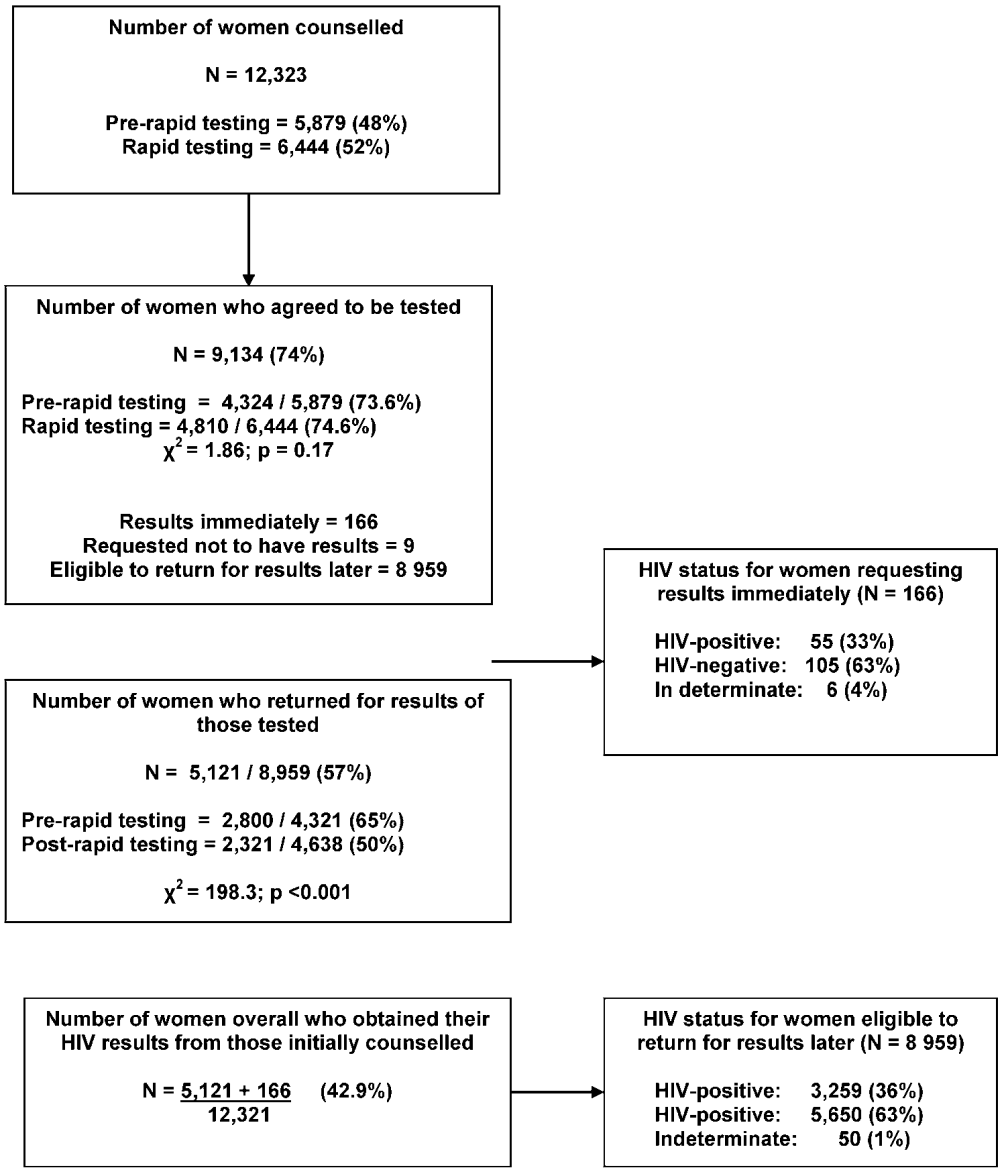
Proportion of women accepting HIV counselling and their HIV results.

Overall, 12,323 pregnant women received HIV counselling; their socio-demographic characteristics are shown in Table 1. Figure 1 shows the proportion of women who accepted testing and those who returned for results. Overall 74% (9,134/12,323) of women accepted testing, of whom 9 did not want their results. In a logistic regression model including maternal education, age and parity, women with secondary school education were less likely to accept testing than those with no education (AOR 0.648; 95% CI 0.54–0.79; p<0.001). Compared to women below 20 years, those aged 21–35 years were less likely to accept testing (AOR 0.762; 95% CI 0.68–0.85; p<0.001); as were those above 35 years (AOR 0.756; 95% CI 0.62–0.93; p<0.01) (Table 2).

**Table 1:** Characteristics of pregnant women undergoing counselling for HIV.

Variable	Women counselled N = 12,323
**Maternal age, years**	
Median (range)	23 (14–53)
14–20	4399 (36%)
21–35	6893 (56%)
>35	975 (8%)
Unknown	56
**Previous pregnancies, n (%)**	
Median (range)	1 (0–15)
**Previous live births, n (%)**	
Median (range)	1 (0–13)
None	5294 (44%)
1–4	6115 (51%)
>5	591 (5%)
Unknown	323
**Highest level of education achieved, n (%)**	
None	758 (6%)
Some primary	4220 (35%)
Some secondary	7015 (59%)
Unknown	330

**Table 2:** Maternal factors associated with agreeing to be tested for HIV (n = 11,818)*.

Variable	N	OR (95%CI)	p	AOR (95%CI)	p
**Education**					
None	739	1.00		1.00	
Primary	4160	0.95 (0.79–1.14)	0.57	0.846 (0.70–1.02)	0.09
Secondary	6919	0.76 (0.64–0.91)	<0.01	0.648 (0.54–0.79)	<0.001
**Number of previous live born**					
None	5217	1.00		1.00	
1–4	6023	0.84 (0.77–0.92)	<0.001	0.915 (0.82–1.02)	0.10
>5	578	0.81 (0.67–0.99)	0.04	0.795 (0.63–1.01)	0.06
**Maternal age (years)**					
14–20	4286	1.00		1.00	
21–35	6647	0.736 (0.67–0.81)	<0.001	0.762 (0.68–0.85)	<0.001
>35	885	0.786 (0.67–0.93)	<0.01	0.756 (0.62–0.93)	<0.01

* n = 11,818 as there were 505 women of the 12,323 who were counselled who had missing values.

Overall, 57% of women who accepted testing returned for their results (Figure 1). Women were more likely to return for results before rapid tests were introduced than after (65% vs. 50%, p<0.001). In logistic regression, including women who accepted testing (n = 8,959), there was a declining trend over time for returning for results: those testing in 2002 (OR 0.61; 95% CI 0.46–0.81; p<0.001), 2003 (OR 0.23; 95% CI 0.18–0.31; p<0.001) and 2004 (OR 0.32; 95% CI 0.24–0.42; p<0.001) were less likely to return for results compared to those testing in 2001. From adjusted and unadjusted analyses, the odds of women returning for their results was not significantly associated with maternal age, education, number of previous live births, or HIV status.

Of the total 12,323 women receiving counselling, 6,444 were counselled after May 2003 when rapid tests were introduced. Of these, 4,810 accepted testing (74.6%) and had both rapid tests (with a definitive result offered) and blood sent for laboratory ELISA testing. 166 requested their results the same day (166/4,810; 3.4%) but the remainder made an appointment to return in two weeks time. Of 12,323 women initially counselled, 42.9% actually received their results (5,121 who returned later, and 166 who received immediate results).

## Discussion

Our 3-stage counselling process enabled us to counsel many women in one day and was well accepted by women and clinic staff. The rates of acceptance of HIV testing were not significantly different before and after the introduction of rapid testing, in line with reports from other studies[8], [11]. Surprisingly, older and more educated women were less likely to accept testing, suggesting the need to specifically target these clients. Despite relatively high rates of testing, many women failed to return for results. After introduction of rapid testing, only 3.4% of women opted for same-day results; a finding contrary to reports from non-PMTCT settings in the States which showed that clients were more likely to receive their results with rapid than conventional tests[12].

Rapid testing may not be appropriate for all individuals, and people's ability and readiness to receive their test result must be respected[6]. There are many possible reasons for the reluctance to accept same-day results and, as this was an observational cohort, and not a randomized controlled trial set up to examine this question, it is not possible to state with certainty what women's reasons were in this context. However, possibilities include: rural women often walk long distances to clinics and have already waited for long periods at the clinic; the option to go home and collect results at their next antenatal appointment seems attractive[11], [21]; women receive a lot of information at their booking visit, may be learning about the PMTCT programme for the first time, and want time to consider their personal risks and support network before accepting results. It is also possible that the counselling process did not evolve appropriately after the introduction of rapid testing, so that counsellors did not yet adequately prepare women for same-day results. Although the counsellors were all trained in the process of rapid testing, the low rates of women who accepted their rapid test results on the same day may indicate that the existing counselling process should have been examined more carefully. In addition, secular changes may have taken place in the community over the period of the study that affected attitudes to counselling and testing, and staff attitudes may have been different at the beginning and end of the study period.

Although rapid testing is available at most antenatal clinics in this province, this does not imply that all women know their status. This could explain some of the failures of the PMTCT programme and the current high rates of peri-partum transmission[22]. Strategies to increase the proportion of women who know their status during pregnancy are urgently required to decrease the unacceptable rates of MTCT of HIV, and to ensure that women with low CD4 counts enter ART programmes at the appropriate times.

Rapid testing has many advantages in poor-resource, high HIV prevalent settings, and is being implemented successfully in many areas of the world. We suggest that more efforts are placed on ensuring high quality counselling, particularly preparing clients for same-day results, and examining barriers to testing and accepting results, rather than abandoning this technique altogether.

## References

[1] Manzi M, Zachariah R, R T (2005). High acceptability of voluntary counseling and HIV testing but unacceptable loss to follow up in a prevention of mother-to-child HIV transmission programme in rural Malawi: scaling up requires a different way of acting.. Trop Med and Int Health.

[2] Guay LA, Musoke P, Fleming T, Bagenda D, Allen M (1999). Intrapartum and neonatal single-dose nevirapine compared with zidovudine for prevention of mother-to-child transmission of HIV-1 in Kampala, Uganda: HIVNET 012 randomised trial.. Lancet.

[3] Moodley D, Moodley J, Coovadia H, Gray G, McIntyre J (2003). A multicenter randomized controlled trial of nevirapine versus a combination of zidovudine and lamivudine to reduce intrapartum and early postpartum mother-to-child transmission of human immunodeficiency virus type 1.. J Infect Dis.

[4] Ekouevi D, Leroy V, Viho I (2004). Acceptability and uptake of a package to prevent mother-to-child transmission using rapid HIV testing in Abidjan, Cote d'Ivoire.. AIDS.

[5] Bajunirwe F, Muzoora M (2005). Barriers to the implementation of programs for the prevention of mother-to-child transmission of HIV: a cross-sectional survey in rural and urban Uganda.. AIDS Research and Therapy.

[6] Galvan F, Brooks R, Leibowitz A (2004). Rapid HIV testing: Issues in Implementation.. AIDS Patient Care and STDs.

[7] Hutchinson P, Mahlalela X (2006). Utilization of voluntary counselling and testing services in the Eastern Cape, South Africa.. AIDS Care.

[8] Kalichman S, Simbayi L (2003). HIV testing attitudes, AIDS stigma and voluntary counseling and testing in a black township in Cape Town, South Africa.. Sexually Transmitted Infections.

[9] Matovu J, Gray R, Makumbi F (2005). Voluntary HIV counseling and testing acceptance, sexual risk behaviour and HIV incidence in Rakai, Uganda.. AIDS.

[10] Pignateli S, Simpore J, Pietra V (2006). Factors predicting uptake of voluntary counseling and testing in a real-life setting in a mother-to-child center in Ougadougou, Burkina Faso.. Trop Med and Int Health.

[11] Thielman N, Chu H, Ostermann J (2006). Cost-effectiveness of free HIV voluntary counselling and testing through a community-based AIDS service organization in Northern Tanzania.. Am J Public Health.

[12] Hutchinson A, Branson B, Kim A (2006). A meta-analysis of the effectiveness of alternative HIV counseling and testing methods to increase knowledge of HIV status.. AIDS.

[13] UNAIDS/WHO (2005). AIDS Epidemic Update.. http://www/unaids.org/epi/2005/doc/report_pdf.

[14] Welz T, Hosegood V, Jaffar S, Batzing-Feigenbaum J, Herbst K (2007). Continued very high prevalence of HIV infection in rural KwaZulu-Natal, South Africa: a population-based longitudinal study.. AIDS.

[15] Coovadia H, Rollins N, Bland R, Little K, Coutsoudis A (2007). Mother-to-child transmission of HIV-1 infection during exclusive breastfeeding: the first six months of life.. Lancet.

[16] Rollins N, Coovadia H, RM B, Coutsoudis A, Bennish M (2007). Pregnancy outcomes in HIV-infected and uninfected women in rural and urban South Africa.. JAIDS.

[17] WHO/UNICEF/UNAIDS (2000). HIV and infant feeding counselling: a training course.

[18] WHO/UNICEF (1993). Breastfeeding counselling: A training course.

[19] KwaZulu Natal Provincial Department of Health Protocol for the Phased Implementation of a Comprehensive Package of Care for the Prevention of Mother to Child Transmission of HIV in KwaZulu Natal..

[20] Tanser F, Hosegood V, Barnighausen T, Herbst K, Nyirenda M (2007). Cohort profile: Africa Centre Demographic Information System (ACDIS) and population-based survey.. int J Epidemiol.

[21] Tanser F, Gijsbertsen B, Herbst K (2006). Modelling and understanding primary health care accessibility and utilization in rural South Africa: An exploration using a geographical information system.. Soc Sci Med.

[22] Rollins N, Little K, Mzolo S, Horwood C, Newell M (2007). Surveillance of mother-to-child prevention programmes at immunisation clinics: the case for universal screening.. AIDS.

